# Automated Step Detection in Inertial Measurement Unit Data From Turkeys

**DOI:** 10.3389/fgene.2020.00207

**Published:** 2020-03-19

**Authors:** Aniek Bouwman, Anatolii Savchuk, Abouzar Abbaspourghomi, Bram Visser

**Affiliations:** ^1^Animal Breeding and Genomics, Wageningen University & Research, Wageningen, Netherlands; ^2^Jheronimus Academy of Data Science (JADS), ’s-Hertogenbosch, Netherlands; ^3^Hendrix Genetics, Boxmeer, Netherlands

**Keywords:** inertial measurement unit, step detection, gait analysis, segmentation, accelerometer

## Abstract

Locomotion is an important welfare and health trait in turkey production. Current breeding values for locomotion are often based on subjective scoring. Sensor technologies could be applied to obtain objective evaluation of turkey gait. Inertial measurement units (IMUs) measure acceleration and rotational velocity, which makes them attractive devices for gait analysis. The aim of this study was to compare three different methods for step detection from IMU data from turkeys. This is an essential step for future feature extraction for the evaluation of turkey locomotion. Data from turkeys walking through a corridor with IMUs attached to each upper leg were annotated manually. We evaluated change point detection, local extrema approach, and gradient boosting machine in terms of step detection and precision of start and end point of the steps. All three methods were successful in step detection, but local extrema approach showed more false detections. In terms of precision of start and end point of steps, change point detection performed poorly due to significant irregular delay, while gradient boosting machine was most precise. For the allowed distance to the annotated steps of 0.2 s, the precision of gradient boosting machine was 0.81 and the recall was 0.84, which is much better in comparison to the other two methods (<0.61). At an allowed distance of 1 s, performance of the three models was similar. Gradient boosting machine was identified as the most accurate for signal segmentation with a final goal to extract information about turkey gait; however, it requires an annotated training dataset.

## Introduction

Locomotion is an important welfare and health trait in turkey production. Impaired locomotion compromises growth and (re)production. Breeding programs tend to record locomotion of selection candidates by scoring the conformation or walking ability by a human expert (Quinton et al., 2011). These scores are repeatable, heritable, and valuable to the breeding program. However, these scores are subjective and labor intensive and require animal handling. Therefore, an objective automated locomotion score would be preferred. Sensor technology seems a promising tool for this task. Additionally, it provides opportunities for repeated measurements of individuals, which could lead to more accurate breeding values.

In recent years, sensor technologies have been introduced in livestock production, and some of them are well suited for objective locomotion scoring like force platforms, cameras, and accelerometers. Force platform systems have been used for locomotion phenotyping in experimental setups (Pastell et al., 2006; Nääs et al., 2010; Maertens et al., 2011; Pluym et al., 2013), but applications on farms are limited because they are expensive and require frequent maintenance. Cameras are upcoming tools in cattle and pigs, with the advantage that they do not disturb the animal (Kashiha et al., 2014; Viazzi et al., 2014; Kuan et al., 2019). However, adding individual animal identification to the image data is a challenge. Accelerometers are widely applied to individual cows and pigs for detecting behavioral changes over time that can indicate signs of estrus and health or welfare impairment, including lameness (e.g., Pastell et al., 2009; Escalante et al., 2013; Tamura et al., 2019). Inertial measurement units (IMUs) are a combination of accelerometer, gyroscope, and sometimes a magnetometer. Besides acceleration, they also measure rotational velocity; together, they can indicate orientation. Human locomotion has been well studied using IMUs, where they are considered as a cost-effective alternative to optical motion systems, which are the golden standard in kinematic analysis in laboratory settings (e.g., Seel et al., 2014; Kluge et al., 2017).

In order to use IMUs for objective evaluation of turkey locomotion, it is essential to describe individual steps by extracting its features. Hence, accurate automated step segmentation of the IMU profile, i.e., defining the start and end point of a single step, is an essential first challenge. Therefore, the aim of this study was to compare different methods for automated step detection from IMU data from turkeys.

## Materials and Methods

### IMU Data

The IMUs used for this study were wireless inertial-magnetic motion trackers (MTw Awinda, XSens Technologies B.V., Enschede, Netherlands). Each IMU contains a triaxial accelerometer, triaxial gyroscope, and triaxial magnetometer. An IMU weighs 16 g and its dimensions are 47 mm × 30 mm × 13 mm. The IMU data were logged to a computer in real time via a receiver.

Data were collected during the standard walkway test applied in the turkey breeding program of Hybrid Turkeys (Hendrix Genetics, Kitchener, Canada). In total, 85 animals were recorded during 1 day. The animals were 20 weeks of age. Two IMUs were attached using Velcro straps, one on each upper leg. Then, the animal was placed in a corridor (∼1.5 m wide) and stimulated to walk in one direction for approximately 5 m. The floor was covered with bedding the animals were familiar with. Because these data were recorded during routine processes, there was little time for the animals to get used to the IMUs around their legs. Occasionally, the animals needed stimulation to start walking or during walking. A person walked along with the animal and waved his hand if needed; when waving was not effective, the animals were tapped on the back or finally pushed.

The recording of the IMU was at 100 Hz and was manually started and stopped; on average, there was 20 s recording material per animal. The IMU output consisted of calibrated time series data for triaxial acceleration, triaxial free acceleration, triaxial angular velocity, and triaxial magnetic field. In addition, orientation data are provided in Euler representation (pitch, roll, yaw), as well as unit quarternions represented by a normalized quaternion *q* = [W X Y Z], with W being the real component and X, Y, Z being the imaginary parts. More information about the output data can be found in Paulich et al. (2018).

### Annotation

The annotation was based on the knowledge that a complete single step can be divided into two stages: (1) from the foot separating from the ground to the foot reaching the highest point, the acceleration at this stage starts to increase until it reaches a maximum value; (2) from the highest point of the foot to the foot hitting the ground, at this stage, the acceleration drops from the maximum value to the minimum value (Wang et al., 2017). In the IMU profile, clear indications of movement can be seen. Knowing that the main actual movement of the animals is walking through the corridor, it is safe to assume these are steps. The location of the steps was annotated by hand for 20 IMU profiles: both leg IMUs of 10 different animals. Plots of acceleration magnitude against time were used to define start and end position of the step. Acceleration magnitude was calculated as $\sqrt{A\,c\,c\,\_\,X^{2}+A\,c\,c\,\_\,Y^{2}+A\,c\,c\,\_\,Z^{2}}$ where Acc_X, Acc_Y, and Acc_Z are the output of the triaxial accelerometer in *X*, *Y*, and *Z* direction, respectively (Wang et al., 2017). Half steps at the beginning or end of a profile were not annotated, as well as insignificant movements (acceleration magnitude <20 m/s^2^). The number of annotated steps per profile ranged between 7 and 15 with an average of 9.9. These ranges were within expectation from turkeys this age walking ∼5 m. In total, 198 steps (sum of annotated steps over 20 profiles) were annotated and available for further analysis. Although more IMU profiles were available, they were not manually annotated, because the manual annotation was too time-consuming. In addition, the 20 IMU profiles resulted in sufficient steps (198) for model development and performance testing (shown below).

Although we use this manual step annotation for model training and performance evaluation, we do not consider it as the truth. The start and end position of the annotated steps are not perfectly accurate, but this was the best we could do with the data at hand. The step annotation allows us to compare performance of the different methods applied and what may cause the observed differences.

### Change Point Detection

Change point is used to denote a significant variation in the probability distribution of time series. Detection of such variations and exact moments when they occurred may be accomplished with the broad family of supervised and unsupervised methods, the extensive overview of which can be found in Aminikhanghahi and Cook (2017). An application of change point detection (CPD) approaches to step segmentation were demonstrated, for example, by Martinez and De Leon (2016). In our study, we applied an unsupervised version of CPD that is called a singular spectrum transformation (SST). The basic idea of SST is that, for each time point, it compares distribution in the interval before the time point and an interval of the same length after the time point and, based on this, assigns a change point score. Comparison of distributions is made by comparing singular spectrums of two trajectory matrices for these consecutive intervals (Aminikhanghahi and Cook, 2017).

We developed the following algorithm based on the SST that was applied to three acceleration signals Acc_X, Acc_Y, Acc_Z:

(a) To each signal, we applied a low-pass filter to reduce the presence of noise. This step was required because SST does not consider the effect of noise on the system (Aminikhanghahi and Cook, 2017).
(b) For each de-noised signal, we applied SST with windows equal to 10 time points and calculated a change point score.
(c) When the change point score was above 5% of the maximum for a given signal, we declared the step; when it was lower, we declared no step.
(d) Final decision was made by majority: if at least two of the three acceleration signals indicated the step, we declared step at that moment.

### Local Extrema Approach

The local extrema approach (LEA) was inspired by the idea that the significant local extrema in a signal should be associated with the changes in the leg movements. Assuming that we have a set of signals, the method can be described by the following procedure:

(a) To each signal (we used Acc_X, Acc_Y, Acc_Z, Gyr_X, Gyr_Y, Gyr_Z, Roll, Pitch, and Yaw here), we applied a low-pass filter to reduce the presence of noise.
(b) With a sliding window equal to 140 time points, we found local minima and local maxima for each signal. Based on the training data, a window of 140 time points ensures there is at least one step in the sliding window, making it the most optimal window to detect local extrema.
(c) From the set of all extrema discovered in step (b), we filtered out those that were within less than 0.5 standard deviations from the surrounding 10 measurements. This step helped us, for example, to get rid of extrema found in the regions where an animal was not moving and no step had occurred.
(d) We combined significant local extrema from all the considered signals into one set. Then, we kept only those significant extrema that were found in more than one signal or those for which there exists at least one other extrema within 0.1 s (10 measurement to the right or 10 to the left). We will refer to such extrema as important extrema.
(e) Based on the detected important extrema and density of their distribution, we created a list of potential intervals that contain steps. For the first local extrema, we formed an interval that starts in that point and has a length of 0.6 s. Then, for the first extrema that has not ended up in that interval, we checked whether it is within 0.12 s to the end of the created interval, and if so, we added it to the interval, consequently updating the interval’s length. We continued this procedure until we could not find a new extrema within 0.12 s to the interval. Then, we recorded the detected interval as potential step interval and repeated the procedure for the next important extrema. The time thresholds of 0.6 and 0.12 s were chosen based on the data to assure that only one step was occurring within an interval. From the annotated data, the average step length was 0.6 s, and there was at least 0.12 s in between two steps.
(f) If, in some potential step, interval distance between the first important extrema to the second important extrema was higher than 0.12 s, we removed the first important extrema from the interval and proceed the check for the next element of the interval.
(g) We filtered out intervals that have duration shorter than 0.2 s, because that is too short to be an actual step. We also filtered out intervals that have two consecutive important extrema that were located more than 0.25 s from each other, to assure a dense set of important extrema that support the evidence of a step. These thresholds were based on the annotation of the steps.
(h) Finally, to avoid false-positive indications, we filtered out intervals within which acceleration magnitude was lower than 11 m/s^2^. Real steps always showed an acceleration magnitude peak higher than 11 m/s^2^; below this value, it may be noise or tremor in the legs.

The obvious drawback of the LEA method is that it depends on a high number of parameters that probably will be different for other species or even another age group of turkeys. While some parameters allow slight deviation from the given numbers, the set of measured signals does not. We found that it is impossible to build successful LEA for step segmentation based only on acceleration signals. The density of received important extrema does not allow to distinguish between parts of the signals that correspond to the step and no step periods.

### Gradient Boosting Machine

We used a gradient boosting machine (GBM) implemented in R version of H2O 3.20.0.8 (Landry, 2018) to predict the time points that are part of steps in IMU profiles. The GBM method is a supervised learning task with the advantage of high performance and interpretable models (Elith et al., 2008). Each individual time point within an annotated step was classified as step and each individual time point outside an annotated step was classified as non-step for model training. To build the GBM model, 60% of the 198 manually annotated steps were used for training (125 steps from 12 IMU profiles), 20% for validation (36 steps from 4 profiles), and 20% as an independent test set (37 steps from four profiles).

Except for magnetic field data, all standard IMU output parameters as described above were used for step prediction, as well as magnitude of acceleration. Each of the 20 parameters were transformed by taking the difference between the actual time point measure and a lag or lead measure. Each time series was lagged by 5 to 10 time points, and led by 5 to 10 time points. The lag/lead was applied to detect significant changes in the profiles, the 5 to 10 time points relates to 0.05 to 0.1 s, which seemed reasonable for significant changes in movement during walking. This resulted in 12 different time series per parameter (e.g., Acc_X_lag5-Acc_X_lag10, Acc_X_lead5-Acc_X_lead10), and a total of 240 predictor variables for the GMB prediction of steps. We ran the GBM model with default settings. The model parameters were as follows: number_of_trees was 50, number_of_internal_trees was 50, model_size_in_bytes was 21040, min_depth was 5, max_depth was 5, mean_depth was 5, min_leaves was 24, max_leaves was 32, and mean_leaves was 28.48. Performance results of the selected GBM model can be found in the Supplementary Material.

The prediction of the step per time point is not very helpful in defining features from a step; hence, step start and end needed to be defined. Based on the predictions per time point, we defined the start and end moment of the steps. Starting from the first time point predicted to be a step, if it had at least 10 consecutive step predictions, it was the starting point of that particular step. The end point of that step was the last time point in the row that was predicted a step. This resulted in some steps being very close to each other, so close that they are likely part of the same (annotated) step. Hence, steps within 10 time points were merged together into one step.

### Performance Assessment

The main goal of step segmentation is to provide subsequences of a signal for the following feature extraction. Therefore, we have two obvious requirements for segmentation methods: (i) to maximize the number of recognized steps; and (ii) precise start and end moments of the recognized steps. To evaluate performance of proposed methods, we applied techniques used in Haji Ghassemi et al. (2018) and Šprager and Jurič (2018). First, we calculated the numbers of true-positive (TP), false-negative (FN), and false-positive (FP) steps for the 198 annotated steps, where TP are steps detected by the method and also labeled manually in the annotation step; FN are steps that were annotated but not detected by the method; FP are steps detected by the method but which were not annotated.

Based on these numbers, we calculated three metrics: precision = *TP*/(*TP* + *FP*) recall = *TP*/(*TP* + *FN*), and F-score = 2(*Precision*×*Recall*)/(*Precision* + *Recall*) (Rijsbergen, 1975). Precision provides punishment for the detected steps that were not annotated; it is equal to one if there are no FP steps. Similarly, recall provides punishment for the annotated steps that were not detected by a method, and is equal to one if there are no FN steps. The F-score is the harmonic mean of precision and recall, and is equal to one if there are no FN or FP steps but decreases with higher number of FP and FN steps (Hand and Christen, 2018).

To be considered as a TP, both the start and end points of detected steps should be within the allowed distance from the corresponding start and end points of the annotated steps. We compared the performance of the different step detection methods at an allowed distance from 0.1 s to 0.5 s and 1 s.

In addition, the average delay per detected start and end point of the corresponding annotated step was calculated for each method. The delay was negative if the detected start or end point was located before the annotated start or end point.

## Results

### Change Point Detection

**FIGURE 1:**
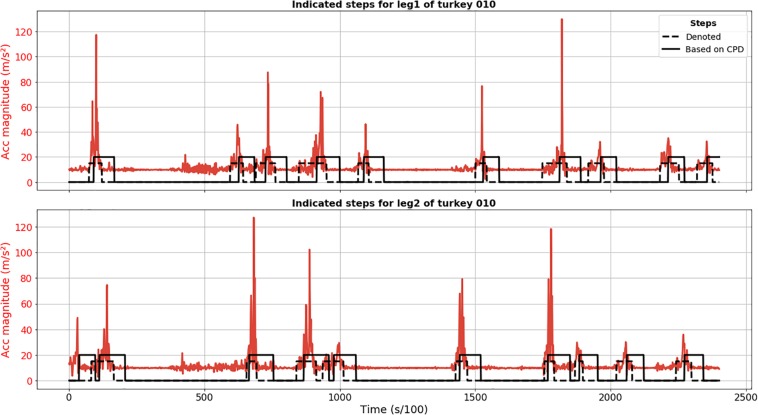
Results of CPD step segmentation for turkey 010 (solid black line) for leg 1 (upper plot) and 2 (lower plot). Acceleration magnitude is plotted in red; dashed black line depicts manually labeled steps.

The proposed approach based on SST method for CPD detected all steps for animal 010 (no FN) and the only FP detection was actually a true step not annotated because its start point might have been before recording (Figure 1). It revealed itself as a quite robust method for step detection. When it comes to the preciseness of the detection of start and end moments, we observed significant delay. The peak of acceleration magnitude is, in some cases, located outside the found interval because of this delay in detection.

### Local Extrema Approach

**FIGURE 2:**
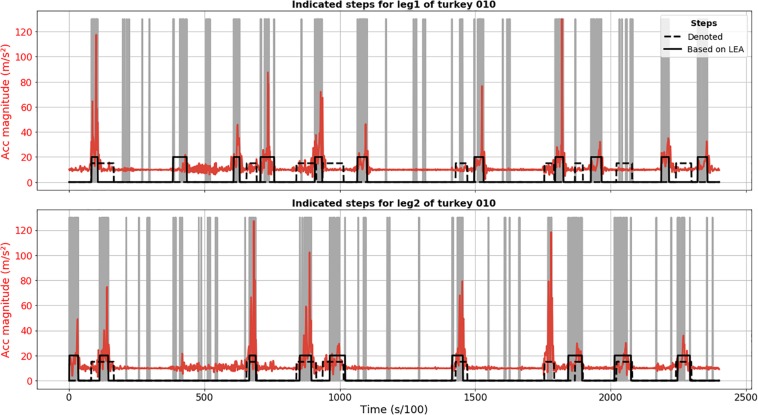
Step segmentation results of LEA for turkey 010 (solid black line) for leg 1 (upper plot) and 2 (lower plot). Acceleration magnitude is plotted in red; dashed black line shows manually labeled steps. Vertical gray lines corresponded to important extrema.

Results of LEA demonstrate less robustness in terms of step detection in comparison to CPD. Figure 2 shows FP detection, like second detected step for leg 1 of turkey 010, as well as FN steps, like sixth annotated step for leg 2. Based on video material, we confirmed that these were truly FP and FN detections.

### Gradient Boosting Machine

**FIGURE 3:**
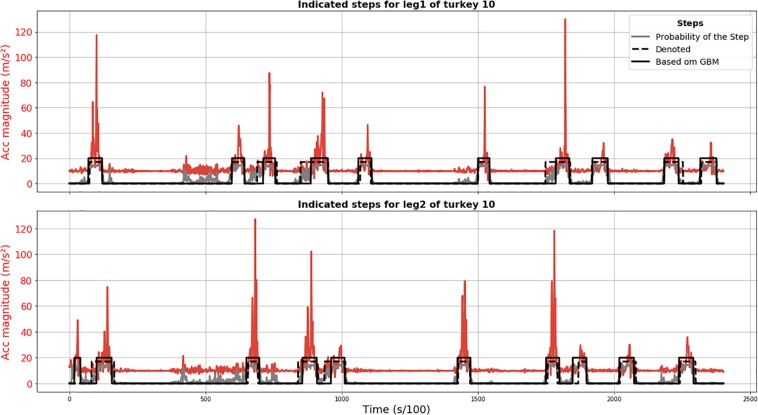
Results of GBM step segmentation for turkey 010 (solid black line) for leg 1 (upper plot) and 2 (lower plot). Acceleration magnitude is plotted in red; dashed black line shows manually labeled steps. The gray line represents the chance of being a step according to the GBM model (×15 for visualization).

Results of the GBM method are plotted in Figure 3 and demonstrate that all annotated steps for animal 010 were detected (no FN) and the only FP detection was actually a true step not annotated because its start point might have been before recording. The position of the steps detected using the GBM model is acceptable, given that the annotation is not perfect either.

### Performance Assessment

Table 1 provides precision, recall, and F-score for the considered methods. All these metrics were calculated based on annotated steps of both legs of four turkeys that were left out of the training of the GBM model. Based on the F-score, GBM performed best at any of the evaluated allowed distances from the annotated start and end points. Somewhat worse result was shown by LEA. For small allowed distances, the performance of CPD was very low. For an allowed distance of 0.1 s, the steps detected by CPD were more than 0.1 s from the start and end point of all annotated steps; hence, none of the steps were declared as TP and precision and recall were zero. However, the performance of CPD considerably increased with an increase of allowed distance.

**TABLE 1:** Performance results of the step detection methods for different allowed distances (in seconds) from the annotated steps.

**Method**	**Metric**	**0.1 s**	**0.2 s**	**0.3 s**	**0.4 s**	**0.5 s**	**1.0 s**
CPD	Precision	0.00	0.01	0.07	0.15	0.49	0.97
	Recall	0.00	0.01	0.07	0.14	0.47	0.91
	F-score	NA^1^	0.01	0.07	0.14	0.48	0.94
LEA	Precision	0.25	0.61	0.74	0.85	0.90	0.95
	Recall	0.23	0.56	0.69	0.78	0.82	0.88
	F-score	0.24	0.58	0.71	0.81	0.86	0.92
GBM	Precision	0.65	0.81	0.87	0.92	0.93	0.97
	Recall	0.67	0.84	0.89	0.95	0.96	1.00
	F-score	0.66	0.82	0.88	0.93	0.95	0.99

*^1^NA = not applicable (due to division by zero).*

Unsatisfying performance of CPD for small values of allowed deviation can be explained by the delay in step detection. There was an average delay for start points of the steps of 0.27 s with a standard deviation of 0.15 s. The average delay for the end points was even higher: 0.47 s with a standard deviation of 0.11 s. This indicated that the delays were not constant; therefore, it is not possible to correct for the delay.

As to delays for LEA and GBM, the corresponding values are much smaller in comparison to CPD. For LEA, average delay in start points was 0.14 s (± 0.15 s), and 0.15 s (± 0.12 s) for end points. For GBM, average delay in start points was 0.09 s (± 0.14 s), and 0.05 s (± 0.05 s) for end points.

## Discussion

The aim of this study was to get accurate start and end position of turkey steps based on IMU data. In terms of step detection in general, all three compared methods were successful, although LEA showed more false detections than CPD and GBM. In terms of precision of start and end point of steps, CPD performed poorly, while GBM was most precise.

Although the data collection was done in a controlled walking test setup, the data are not as clean as experimental setups with humans or trained animals like horses (Pfau et al., 2005). For example, some animals were hesitating and needed stimulation, after which they took a number of short fast steps before continuing at a steady pace. This imposed some difficulties in the annotation of the data, and also hampers accurate step segmentation. It is, however, realistic data, and therefore more diverse and comprehensive compared to experimental setups. This will aid in the development of an algorithm applicable to data retrieved in, for instance, group housing. In addition, it also implicates that the detected steps may need further filtering (e.g., based on standard deviation of certain step features within an individual) depending on the purpose of using the automatically detected steps.

There was no golden standard system applied next to the IMU sensor. In experimental settings, optical motion tracking systems are often used as golden standard to test the IMU performance (e.g., Seel et al., 2014; Kluge et al., 2017; Bosch et al., 2018). Here, we used subjective annotation by a person to define the step start and end positions in the IMU profiles knowing that the animals underwent a walking test. In general, we trust that the annotated steps are true steps and were able to check doubtful cases with video material. However, the exact start and end point are debatable, and might be somewhat different if annotated again or by a different person. Therefore, we showed performance results of the methods for different allowed distances to the annotated steps. Also, steps at the beginning or end of the profile were not annotated because the start or end point was unclear. All three methods detected such steps, but because they were not annotated, they showed up as FP steps in the performance assessment. Although the annotation was suboptimal for evaluation of accurate step segmentation, the results show the potential of each method applied along with their drawbacks.

The main problem of CPD was the inconsistency in delayed detection of start and end point of steps. The delay inhibits accurate extraction of step features describing the locomotion. For example, the maximum acceleration magnitude that might be an important feature is often outside the detected steps. Such results make this method inappropriate for step segmentation with the final goal to extract features that thoroughly describe the turkey gait. However, in general, all annotated steps were detected; therefore, CPD is an appropriate method if exact position is not relevant, for instance for step counting.

As it was demonstrated, LEA does not have problems with the delay. However, it is outperformed by GBM. One possible reason might be that LEA is an unsupervised method and was not trained on the annotated steps that have somewhat subjective nature as they are only an approximation of the truth. LEA has the advantage that it does not need pre-annotation and we believe that with some optimization of parameters, it can be applied for other species as well.

In contrast to the LEA and CPD, the GBM method is a supervised learning method that requires an annotated dataset to train the model. Annotating a dataset is very time-consuming; however, our results showed that the GBM model can be trained on limited annotated data (i.e., 198 steps) with good results for our specific problem. This makes it worthwhile to invest in annotation. We used the GBM as it is state-of-the-art implementation of a powerful classification algorithm, but that does not reject the possibility that some other methods of classification may work with similar level of performance. It would be interesting to see how the trained GMB model performs on IMU data from other species. For optimal performance, it might require a species-specific annotated dataset to build a species-specific GBM model.

We should admit that methods for step detection and segmentation of gait signals are not limited to those evaluated here. There exists a vast number of approaches that use local structure similarly to LEA and, if possible, cyclicity in gait sequences (Derawi et al., 2010; Hundza et al., 2014). The most advanced, for example, presented in Derawi et al. (2010), apply such techniques like dynamic time warping. Another interesting possibility might be represented by clustering. While it does not require pre-annotations, it needs a carefully prepared set of features.

## Conclusion

In this paper, we compared three approaches for segmentation of turkey gait sequences obtained with IMU sensors. CPD is commonly used for this purpose; the LEA was newly developed based on characteristics of the data, while the GBM is an advanced machine learning classification algorithm. We have found that the GBM shows the best performance even for little allowed deviation for the annotated steps. Performance of LEA is somewhat worse. Significant inconsistent delay for start and end point detection makes CPD inappropriate for detailed gait analyses. GBM can be applied for signal segmentation with the final goal to extract information about turkey gait; however, it requires an annotated training dataset.

## Data Availability Statement

The datasets for this article are not publicly available because data is intellectual property of Hendrix Genetics. Requests to access the datasets should be directed to BV, bram.visser@hendrix-genetics.com.

## Ethics Statement

Ethical review and approval was not required for the animal study because The Animal Welfare Body (AWB) of Wageningen Research decided ethical review was not necessary because the applied units were low in weight (<1% of body weight), the units were attached for less than one hour, the animal is not isolated in the corridor and more or less familiar with the corridor.

## Author Contributions

All authors contributed to conception and design of the study, and read and approved the submitted version of manuscript. AB, AS, and AA performed the statistical analysis. AB and AS wrote the manuscript.

## Conflict of Interest

BV and AS were employed by the company Hendrix Genetics. The remaining authors declare that the research was conducted in the absence of any commercial or financial relationships that could be construed as a potential conflict of interest.

## References

[B1] Aminikhanghahi S. Cook D. J. (2017). A survey of methods for time series change point detection. *Knowl. Inform. Syst.* 51 339–367. 10.1007/s10115-016-0987-z 28603327 PMC5464762

[B2] Bosch S. Serra Bragança F. Marin-Perianu M. Marin-Perianu R. Van der Zwaag B. J. Voskamp J. (2018). EquiMoves: a wireless networked inertial measurement system for objective examination of horse gait. *Sensors* 18:850. 10.3390/s18030850 29534022 PMC5877382

[B3] Derawi M. O. Bours P. Holien K. (eds) (2010). “Improved cycle detection for accelerometer based gait authentication,” in *Proceedings of the 2010 Sixth International Conference on Intelligent Information Hiding and Multimedia Signal Processing*, Washington, DC.

[B4] Elith J. Leathwick J. R. Hastie T. (2008). A working guide to boosted regression trees. *J. Anim. Ecol.* 77 802–813. 10.1111/j.1365-2656.2008.01390.x 18397250

[B5] Escalante H. J. Rodriguez S. V. Cordero J. Kristensen A. R. Cornou C. (2013). Sow-activity classification from acceleration patterns: a machine learning approach. *Comput. Electr. Agricult.* 93 17–26. 10.1016/j.compag.2013.01.003

[B6] Haji Ghassemi N. Hannink J. Martindale C. F. Gaßner H. Müller M. Klucken J. (2018). Segmentation of gait sequences in sensor-based movement analysis: a comparison of methods in Parkinson’s Disease. *Sensors* 18:145. 10.3390/s18010145 29316636 PMC5796275

[B7] Hand D. Christen P. (2018). A note on using the F-measure for evaluating record linkage algorithms. *Stat. Comput.* 28 539–547. 10.1007/s11222-017-9746-6

[B8] Hundza S. R. Hook W. R. Harris C. R. Mahajan S. V. Leslie P. A. Spani C. A. (2014). Accurate and reliable gait cycle detection in Parkinson’s Disease. *IEEE Trans. Neural Syst. Rehabil. Eng.* 22 127–137. 10.1109/TNSRE.2013.2282080 24158491

[B9] Kashiha M. A. Bahr C. Ott S. Moons C. P. H. Niewold T. A. Tuyttens F. (2014). Automatic monitoring of pig locomotion using image analysis. *Livestock Sci.* 159 141–148. 10.1016/j.livsci.2013.11.007

[B10] Kluge F. Gaßner H. Hannink J. Pasluosta C. Klucken J. Eskofier B. M. (2017). Towards mobile gait analysis: concurrent validity and test-retest reliability of an inertial measurement system for the assessment of spatio-temporal gait parameters. *Sensors* 17:1522. 10.3390/s17071522 28657587 PMC5539856

[B11] Kuan C. Y. Tsai Y. C. Hsu J. T. Ding S. T. Lin T. T. (2019). “An imaging system based on deep learning for monitoring the feeding behavior of dairy cows,” in *Proceedings of the 2019 ASABE Annual International Meeting* (St. Joseph, MI: ASABE).

[B12] Landry M. (2018). *Machine Learning With R and H2O*, 7th Edn. Mountain View, CA: H2O.ai, Inc. Available at: https://www.h2o.ai/wp-content/uploads/2018/01/RBooklet.pdf (accessed March 9, 2020).

[B13] Maertens W. Vangeyte J. Baert J. Jantuan A. Mertens K. C. De Campeneere S. (2011). Development of a real time cow gait tracking and analysing tool to assess lameness using a pressure sensitive walkway: the GAITWISE system. *Biosyst. Eng.* 110 29–39. 10.1016/j.biosystemseng.2011.06.003

[B14] Martinez M. De Leon P. L. (2016). *Unsupervised Segmentation and Labeling for Smartphone Acquired Gait Data.* Arizona: International Foundation for Telemetering.

[B15] Nääs I. A. Paz I. C. D. L. A. Baracho M. Menezes A. G. Lima K. A. O. Bueno L. G. F. (2010). Assessing locomotion deficiency in broiler chicken. *Sci. Agricola* 67 129–135. 10.1590/s0103-90162010000200001

[B16] Pastell M. Aisla A.-M. Hautala M. Poikalainen V. Praks J. Veermäe I. (2006). Contactless measurement of cow behavior in a milking robot. *Behav. Res. Methods* 38 479–486. 10.3758/bf03192802 17186758

[B17] Pastell M. Tiusanen J. Hakojärvi M. Hänninen L. (2009). A wireless accelerometer system with wavelet analysis for assessing lameness in cattle. *Biosyst. Eng.* 104 545–551. 10.1016/j.biosystemseng.2009.09.007

[B18] Paulich M. Schepers M. Rudigkeit N. Bellusci G. (2018). *Xsens MTw Awinda: Miniature Wireless Inertial-Magnetic Motion Tracker for Highly Accurate 3D Kinematic Applications.* Available at: https://www.xsens.com/hubfs/3446270/Downloads/Manuals/MTwAwinda_WhitePaper.pdf (accessed September 26, 2019).

[B19] Pfau T. Witte T. H. Wilson A. M. (2005). A method for deriving displacement data during cyclical movement using an inertial sensor. *J. Exp. Biol.* 208:2503. 10.1242/jeb.01658 15961737

[B20] Pluym L. M. Maes D. Vangeyte J. Mertens K. Baert J. Van Weyenberg S. (2013). Development of a system for automatic measurements of force and visual stance variables for objective lameness detection in sows: SowSIS. *Biosyst. Eng.* 116 64–74. 10.1016/j.biosystemseng.2013.06.009

[B21] Quinton C. D. Wood B. J. Miller S. P. (2011). Genetic analysis of survival and fitness in turkeys with multiple-trait animal models 1. *Poult. Sci.* 90 2479–2486. 10.3382/ps.2011-01604 22010232

[B22] Rijsbergen C. J. V. (1975). *Information Retrieval.* London: Butterworths.

[B23] Seel T. Raisch J. Schauer T. (2014). IMU-Based joint angle measurement for gait analysis. *Sensors* 14 6891–6909. 10.3390/s140406891 24743160 PMC4029684

[B24] Šprager S. Juriè M. B. (2018). Robust stride segmentation of inertial signals based on local cyclicity estimation. *Sensors* 18:1091. 10.3390/s18041091 29617340 PMC5948565

[B25] Tamura T. Okubo Y. Deguchi Y. Koshikawa S. Takahashi M. Chida Y. (2019). Dairy cattle behavior classifications based on decision tree learning using 3-axis neck-mounted accelerometers. *Anim. Sci. J.* 90 589–596. 10.1111/asj.13184 30773740

[B26] Viazzi S. Bahr C. Van Hertem T. Schlageter-Tello A. Romanini C. E. B. Halachmi I. (2014). Comparison of a three-dimensional and two-dimensional camera system for automated measurement of back posture in dairy cows. *Comput. Electr. Agricult.* 100 139–147. 10.1016/j.compag.2013.11.005

[B27] Wang Y. Vasilakos A. V. Jin Q. Zhu H. (2017). *Device-to-Device based Proximity Service: Architecture, Issues, and Applications.* Boca Raton, CA: CRC Press.

