# Supplementary material for: Automated Step Detection in Inertial Measurement Unit Data From Turkeys
Source: Front Genet. 2020 Mar 19;11:207. doi: 10.3389/fgene.2020.00207 (PMC7096551; doi:10.3389/fgene.2020.00207)
Supplement: Supplementary file 1 [file Table_1.docx]

Supplementary Material

# Supplementary Tables

Supplementary Table 1 shows the performance results of the GBM model on the validation and test set, as well as a 5 fold cross validation. The AUC was 0.97 for the test set. The 5 most informative variables were OriInc_q0_lag10, accmag_lag10, FreeAcc_Z_lag7, OriInc_q0_lag8, Free_Acc_Z_lag8. This indicates that i) lag variables were more relevant than lead variables; ii) lags 7-10 were more relevant than the smaller lags; iii) real normalized quarternion was important as well as acceleration magnitude and free acceleration in Z-axis. The confusion matrix for the test set using the trained GBM model is given in Supplementary Table 2 and shows that the overall error of step prediction of each timepoint is 0.09. However, the errors in prediction are partly due to annotation errors. Therefore, the actual error rate might even be lower than 0.09.

**Supplementary Table 1.** Performance results of the GBM model for the validation and independent test set

| Metric | Training set | Validation set | Test set |
| --- | --- | --- | --- |
| MSE | 0.037 | 0.077 | 0.069 |
| RMSE | 0.193 | 0.278 | 0.262 |
| LogLoss | 0.138 | 0.252 | 0.231 |
| Mean Per-Class Error | 0.063 | 0.118 | 0.092 |
| AUC | 0.989 | 0.956 | 0.970 |
| Gini impurity | 0.978 | 0.912 | 0.940 |

**Supplementary Table 2.** Confusion matrix for the results of the GBM model on the test set

|  | Predicted | |  |  |
| --- | --- | --- | --- | --- |
| Annotated | None | Step | Error | Rate |
| None | 4550 | 363 | 0.073886 | 363/4913 |
| Step | 250 | 2031 | 0.109601 | 250/2281 |
| Totals | 4800 | 2394 | 0.085210 | 613/7194 |
